# Revealing *Escherichia coli* type II l-asparaginase active site flexible loop in its open, ligand-free conformation

**DOI:** 10.1038/s41598-021-98455-1

**Published:** 2021-09-23

**Authors:** Maristella Maggi, Massimiliano Meli, Giorgio Colombo, Claudia Scotti

**Affiliations:** 1 Unit of Immunology and General Pathology, Department of Molecular Medicine, University of Pavia, Via Adolfo Ferrata, 9, 27100 Pavia, Italy; 2 Istituto di Scienze e Tecnologie Chimiche “Giulio Natta”-SCITEC, Centro Nazionale Ricerche (CNR), Milan, Italy; 3 Department of Chemistry, University of Pavia, Pavia, Italy

**Keywords:** Structural biology, X-ray crystallography, Cancer therapy, Chemotherapy, Enzyme mechanisms

## Abstract

Since 1993, when the structure of *Escherichia coli* type II l-asparaginase (EcAII) in complex with l-aspartate was firstly reported, many structures of the wild type and mutated enzyme have been deposited in the Protein Data Bank. None of them report the full structure of the monomer in its ligand-free, open conformation, mainly because of the high dynamic and flexibility of the active site flexible loop. Here we report for the first time the structure of EcAII wild type in its open conformation comprising, for at least one protomer, clear electron density for the active site flexible loop (PDB ID: 6YZI). The structural element is highly mobile and it is transposed onto the rigid part of the active site upon substrate binding to allow completion of the enzyme catalytic center, thanks to key residues that serve as hinges and anchoring points. In the substrate binding pocket, several highly conserved water molecules are coordinated by residues involved in substrate binding, comprising two water molecules very likely involved in the enzyme catalytic process. We also describe, by molecular dynamics simulations, how the transposition of the loop, besides providing the proximity of residues needed for catalysis, causes a general stabilization of the protein.

## Introduction

l-Asparaginases are amidohydrolases (EC 3.5.1.1) found in almost all living organisms. The bacterial enzyme is capable of deaminating l-asparagine and, with reduced efficiency, l-glutamine, to their corresponding acidic forms releasing ammonia^1^. Bacterial type II l-asparaginases are of great interest because of their use in the treatment of Acute Lymphoblastic Leukemia (ALL). *Escherichia coli* type II l-asparaginase (EcAII), in its native (Spectrila^®^) or PEGylated form (ONCASPAR^®^), is used as first line drug for the treatment of ALL^1^. Its chemotherapeutic effect derives from removal of exogenous l-asparagine so that Asparagine Synthetase (AS) deficient leukemia cells are lead to death^2^. Asparaginase-based therapy has a high therapeutic success rate mainly in pediatric patients (80–90% of 5-years event free survival), but the therapy is often accompanied by toxicity and side effects that, in the most severe cases, can lead to therapy interruption^3,4^. The most frequent side effect is the development of immune reactions to the exogenous protein, that can lead to local or systemic symptoms up to anaphylactic shock. Other side effects linked to l-asparaginase-based therapy are of metabolic origin, affecting mainly liver, pancreas and the central nervous system (CNS)^5^. Very likely, these classes of side effects are secondary to removal of circulating l-asparagine and, above all, l-glutamine, which can impair protein synthesis also in healthy tissues with high-transcriptional rate, like liver and pancreas^2^. Moreover, accumulation of ammonia released upon asparagine and glutamine hydrolysis can greatly impair CNS function^6^. Another aspect to consider for l-asparaginase treatment is the drug functional stability after administration. High levels of l-asparaginase activity in patients’ blood are desirable to reach the therapeutic effect, mainly during induction therapy. However, in some cases, circulating l-asparaginase levels quickly drop-off after administration, either because of binding to inactivating antibodies or because of degradation^7,8^.

Accurate structural studies on the protein can help in deeply understanding the molecule weak points that can be tackled in order to improve on its pharmacological properties. The first EcAII structure was described in 1993 by Swain et al.^9^ and was solved in complex with the product l-aspartate (l-Asp). The functional protein is a homotetramer and each monomer (A, B, C and D) consists of two α/β domains connected by a ca. 21 amino acid-long random-coil. Monomers acquire two distinct sets of contacts in the tetrameric setting, the more extended one being between protomers A and C, on one side, and B and D, on the other. These homodimers describe the functional unit of asparaginases, namely the so-called “intimate dimers”. The second set of contacts, less extended, describes the “distant dimers”, represented by protomers A and B, on one side, and C and D, on the other. The overall structure of l-asparaginases presents an internal 222 symmetry^9,10^.

The molecule includes 4 active sites, buried at the intimate dimer interface and mainly built by residues located in each protomer N-domain, as the companion C-domain of the intimate dimer contributes only one residue to the catalytic centre of the enzyme (E283′)^11^. Asparaginase catalytic mechanism consists of two subsequent nucleophile attacks interspersed by an acyl-intermediate^10,12,13^. Two highly conserved threonines, T12 and T89 in EcAII, and a water molecule are the main player in the formation of the tetrahedral intermediates that precede and follow the covalent acyl-intermediate state^11,12,14–16^. Each active site consists of two parts: a rigid one, mainly involved in substrate binding, and a flexible one, carrying two of the six residues involved in catalysis. Upon substrate binding, the flexible loop closes upon the binding pocket, so that all the catalytically relevant residues fall into close proximity^17^. Given the high flexibility of this structural element, none of the deposited *E. coli* type II wild type asparaginase structures that has been solved in a ligand-free form had electron density in this region, and, as a consequence, complete structural data on the APO structure of this enzyme are, to our knowledge, not yet available. Recently, two structures of ligand-free *E. coli* type II l-asparaginase have been deposited in the Protein Data Bank with identification code 6V23^12^ and 6EOK^18^, but both lack a large part of the catalytically relevant flexible loop (missing residues: 23–37 and 15–53, respectively).

Here, we report for the first time the structure of *E. coli* type II asparaginase in its ligand-free, open conformation (APO-EcAII) at 1.6 Å resolution with clear electron density for at least one protomer active site flexible loop, comprising key residues involved in the enzyme catalysis.

## Results and discussion

### Overall structure description

The asymmetric unit (ASU) comprised 4 monomers named A, B, C, and D; chains A and B and chains C and D are associated into separated intimate dimers, each representing the functional unit of l-asparaginases. The tetrameric biological unit used for comparison to other EcAII structures was rebuilt using Pymol^19^ and applying the crystallographic C2 symmetry operators. It included two copies of the CD dimer, < one from one ASU and one (namely, CʹDʹ) from an adjacent ASU (Supplementary Fig. 1).

Conventionally, l-asparaginase intimate dimers are described as AC and BD, respectively. However, for consistency with the deposited PDB designation of the intimate dimers present in the ASU, in the present paper we will refer to the monomers that compose one intimate dimer as C and D, respectively.

The overall fold of APO-EcAII is identical to the one described for l-Asp-bound wild type structures (PDB IDs: 1NNS^20^ and 3ECA^9^). Each monomer comprises two main α/β domains connected by a 21 amino acid long coiled region. The larger N-domain consists of 10 β-strands and 4 α-helices, the smaller C-domain of 4 β-strands and 4 α-helices^9^.

### Conformations of the active site flexible loop

**Figure 1:**
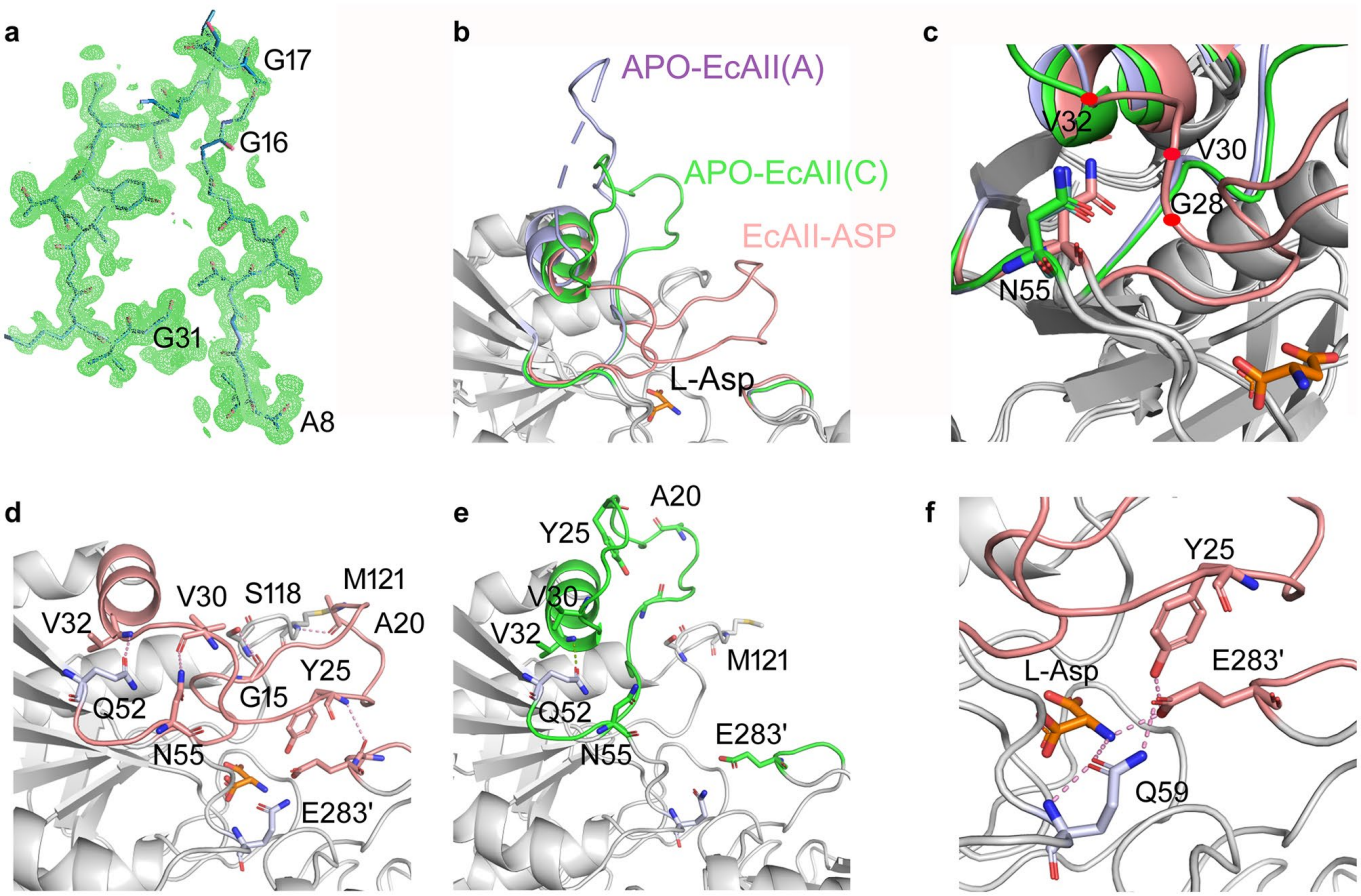
(**a**) Electron density (e.d.) of chain C active site flexible loop (8–31). E.d. (2Fo-Fc map) is shown in green at 0.273 e/Å^3^—7 r.m.s.d., residues as ball and stick. The fragment starting and ending residues are labeled, as well as residues G16 and G17, which showed poor e.d. and were modeled with 0 occupancy. (**b**, **c**) Superposition of the catalytic region of APO-EcAII protomer A (purple, PDB ID: 6IZY) and C (green) and EcAII-ASP (pink, PDB ID: 1NNS). The l-Asp ligand is represented as sticks. In (**c**) only regions different in the three structures are colored. (**d**) EcAII-ASP flexible loop anchoring points. Residues involved in hydrogen bonds are represented as sticks. (**e**) APO-EcAII (protomer C) flexible loop anchoring points. Residues involved in loop anchoring in the l-Asp-bound structure are represented as sticks for comparison. (**f**) Triangular network between intimate dimer residues relevant for catalysis in the EcAII-ASP structure.

The active site flexible loop (residues 11–31) involved in enzyme catalysis is comprised between the β1 strand and the α1 helix in the N-domain. The electron density (e.d.) of residues 16–30 in chain B and of residues 16–31 in chain D was unavailable, which was consistent with most of the available structures for l-asparaginase present in the PDB. However, a clear trace of e.d. was visible for the whole loop in chain C, though with a slightly lower quality for residues G16 and G17 (Fig. 1a and Supplementary Fig. 2b), and for most of it in chain A (missing residues: 25–29).

Figure 1b shows the well-known closed conformation of the loop in the l-Asp-bound form of EcAII (EcAII-ASP, pink, PDB ID: 1NNS) and the open conformations here reported for the APO form (chains A and C in purple and green, respectively) after structure superposition by Pymol^19^.

Residues A8 and V32, located at the edges of the flexible loop, serve as hinge points for the structural changes occurring in the intervening sequence upon substrate binding, allowing opening and closing of the flexible loop with respect to the rigid part of the active site. Compared to the closed form, the active site flexible loop has been captured in different, open orientations in two chains of the new crystal structure. In chain C, it is partially bent towards the α1 helix and its residues S23 and G28 form contacts with residues A38 and N34, respectively (Fig. 1c and Supplementary Fig. 2a). As a consequence, its vertex (i.e., T21) forms a 45.7° angle with respect to the corresponding residue in EcAII-ASP (Supplementary Fig. 3a,b). In chain A, the loop is even more open, and residue T21 forms a 74.5° angle with the position of the same residue of the l-Asp-bound monomer (Supplementary Fig. 3b). In this case, though S23 is solvent exposed and does not interact with A38, both G28 and G16 from the active site flexible loop contact the α1 helix at N34. These three different orientations (one closed and two open) represent different physiological conformations of the active site flexible loop, being a highly mobile element of l-asparaginases, is frozen in place by different interactions in the crystal packing. In the case of our molecule, the loop of monomer C interacts with residues G16, G17 and A38 from monomer A of the same ASU and with T119, S120, M121 from monomer B of a nearby ASU. Interestingly, there are no other APO-structures with the same C2 symmetry of our molecule in the PDB database. The other two available APO-EcAII structures, (PDB IDs: 6V23 and 6EOK), have symmetry I222 and P2_1_2_1_2_1_, respectively. The latter includes a zinc ion close to the binding site and we do not consider an appropriate match for a comparison. Superposition of the monomeric ASU from 6V23 onto monomer C of 6YZI shows that the interactions we observe, needed for the stabilization of the loop, are prevented in the former by a displacement of the neighboring monomers corresponding to chain A of 6YZI (Supplementary Fig. 4 shows only one of them for clarity).

### Structural reorganization upon substrate binding: APO-EcAII vs. EcAII-ASP

Beyond the very mobile flexible loop, even residues 32–38 and 53–55 in the N-domain and residue 283 in the C-domain show a slightly different conformation in the l-Asp-bound versus the APO structure (Fig. 1c, colored elements).

Residues 32–38 belong to the α1 helix, the first structural element located immediately after the active site flexible loop, which undergoes a mild structural reorganization when the loop closes upon the rigid part of the active site. Nevertheless, V32 role as a hinge between the protomer mobile and rigid parts is confirmed, as contacts between V32 main chain and Q52 side chain are maintained in both APO and l-Asp-bound structures (Fig. 1d,e).

Important anchoring points for the loop in its closed conformation are provided by N55 and E283′. Regarding the former, residues 53–55 belong to the β2 strand. The main change in contacts occurring here upon substrate binding impacts on N55, that is free in the unbound form and, instead, becomes engaged with the main chains of G28 and V30, part of the active site flexible loop, in the l-Asp-bound structure. Other anchoring points involve three further residue pairs, where the first partner belongs to the active site loop and the second is located outside it: G15–S118, A20–M121 and Y25–E283′, which are not present in the APO-structure (Fig. 1d,e).

E283′, which has no contacts within the intimate dimer in the APO structure (Fig. 1e), is the only residue involved in substrate binding that belongs to the companion protomer in the C-domain of the intimate dimer and represents an anchoring point of the loop in its closed conformation. Beside interacting with the l-Asp ligand N atom in the bound form, in fact, E283′ creates a triangular network with residues Y25 and Q59 (Fig. 1f). Thus, Y25, which is part of the active site flexible loop, interacts with the catalytic residue T12 and with E283′ in the l-Asp-bound structure. The structure of the Y25F mutant in complex with l-Asp (PDB ID: 1HO3^21^) shows the loop in a closed position with the catalytically relevant T12 in a location superposable to the corresponding residue in the wild type enzyme. In this mutant, F25 has no contacts with E283′ that, in turn, is not interacting even with Gln59. Catalytically, the Y25F mutant has a greatly reduced k_cat_, but preserved K_m_^22^, pointing to a more relevant role of Y25 in the reaction chemistry than in active site loop closing. Indeed, the contact network created upon substrate binding and involving residues Y25–Q59–E283′ has a relevant role in enzyme catalysis as showed by several mutagenesis studies on Q59^22–24^, and the consequent interaction between Q59 and E283′ may help in stabilizing the loop in the closed conformation needed for proper catalysis. As well as the Q59 mutants, E283G and E283V EcAII mutants have reduced l-asparaginase efficiency (55 and 63 times lower, respectively)^22^. In *H. pylori* type II l-asparaginase E289, corresponding to EcAII E283 residue, replacement with an Ala also resulted in the reduction of l-asparaginase activity and abolishment of l-glutaminase activity^24^.

### Catalytic loops

**Figure 2:**
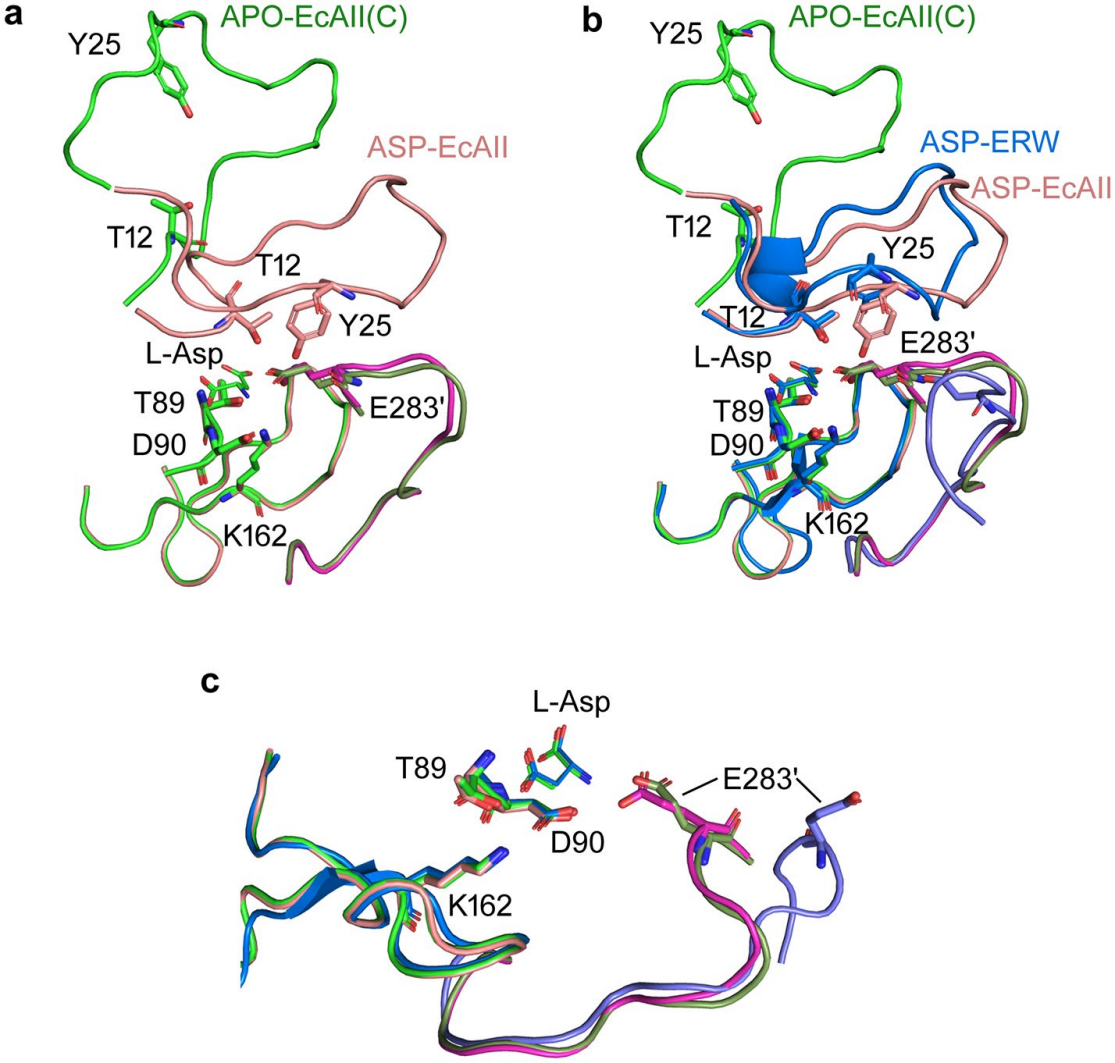
Superposition of APO-EcAII (light or dark green, PDB ID: 6YZI) and EcAII-ASP (light or dark pink, PDB ID: 1NNS) (**a**), and of APO-EcAII (light or dark green, PDB ID: 6YZI) and ErAII-ASP (blue or marine, PDB ID: 5F52) (**b**) catalytic loops. Residues belonging to catalytic triads are represented as sticks. Structural elements belonging to the companion protomer in the intimate dimer are colored in dark for each structure. (**c**) Detail of E283′ different localization in ErAII-ASP.

**Figure 3:**
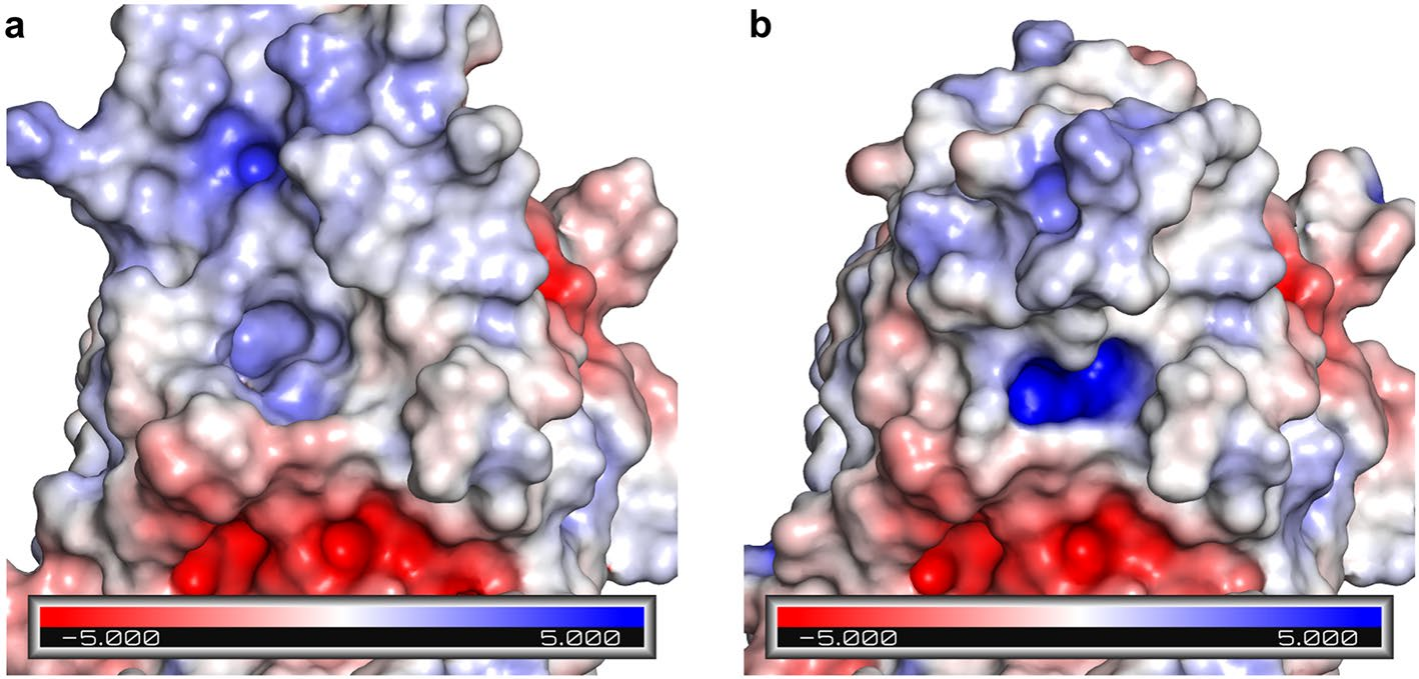
Electrostatic potential surface (EPS) of APO-EcAII (PDB ID: 6YZI) (**a**) and EcAII-ASP (PDB ID: 1NNS) (**b**). The focus is on the monomer N-terminal substrate binding pocket, central in each panel. Unit of measurement of electrostatic potential: kbT/e.

Since its initial description, l-asparaginase catalytic activity has been related to two catalytic triads, each comprising a highly conserved threonine that acts as direct or indirect nucleophile during the enzyme two-step catalysis^12^. Threonine is a weak nucleophile and needs the intervention of a strong basis to be activated: in EcAII the two catalytic triads include residues T12-Y25-E283′ and T89-K162-D90. All six residues lay on unstructured portions of the protomer (Fig. 2a), in particular T12 and Y25 belong to the active site flexible loop (residues 11–31), T89 and D90 belong to the 88–91 loop, located between the β3 strand and the α3 helix, K162 belongs to the 157–178 loop, located between the β5 strand and the β6 strand, and E283′ belongs to the 272–285 loop, located between the α7 helix and the α8 helix in the protomer C-domain. Apart from the active site flexible loop, all the other structural elements and the catalytically relevant residues of the APO-EcAII are superposable to the corresponding elements in the EcAII-ASP structure. Notably, also loop 272–285, belonging to the companion monomer in the catalytically functional intimate dimer, has a position similar to the enzyme in its closed conformation. If compared to the corresponding structural element in the high glutaminase activity *Erwinia chrysanthemi* l-asparaginase (ErAII)^15^, the 272–285 loop is the only active site structural element that has a different organization in the two enzymes, which can explain the different capability of the two enzymes to process l-glutamine as a substrate (Fig. 2b,c). The oxyanion hole described for all the l-asparaginase structures solved in complex with both L-Asp and l-Glu products^10^, which is partially built by residues contributed by the loop in its closed position, is not evident in the new APO-EcAII structure here described. Comparison of the electrostatic properties at the protein surface in the substrate binding pocket between APO-EcAII and EcAII-ASP (Fig. 3a,b, respectively) suggests that most of the positive electrostatic potential in the hole is contributed by residues belonging to the active site flexible loop, in particular the N atoms of residues G11-T12-I13 (see “Materials and methods” section for calculation details). Therefore, translation of the active site flexible loop towards the substrates binding pocket not only contributes important residues for catalysis, but is also necessary to create the proper electrostatic environment for the reaction to happen.

### Water displacement in the binding pocket

**Figure 4:**
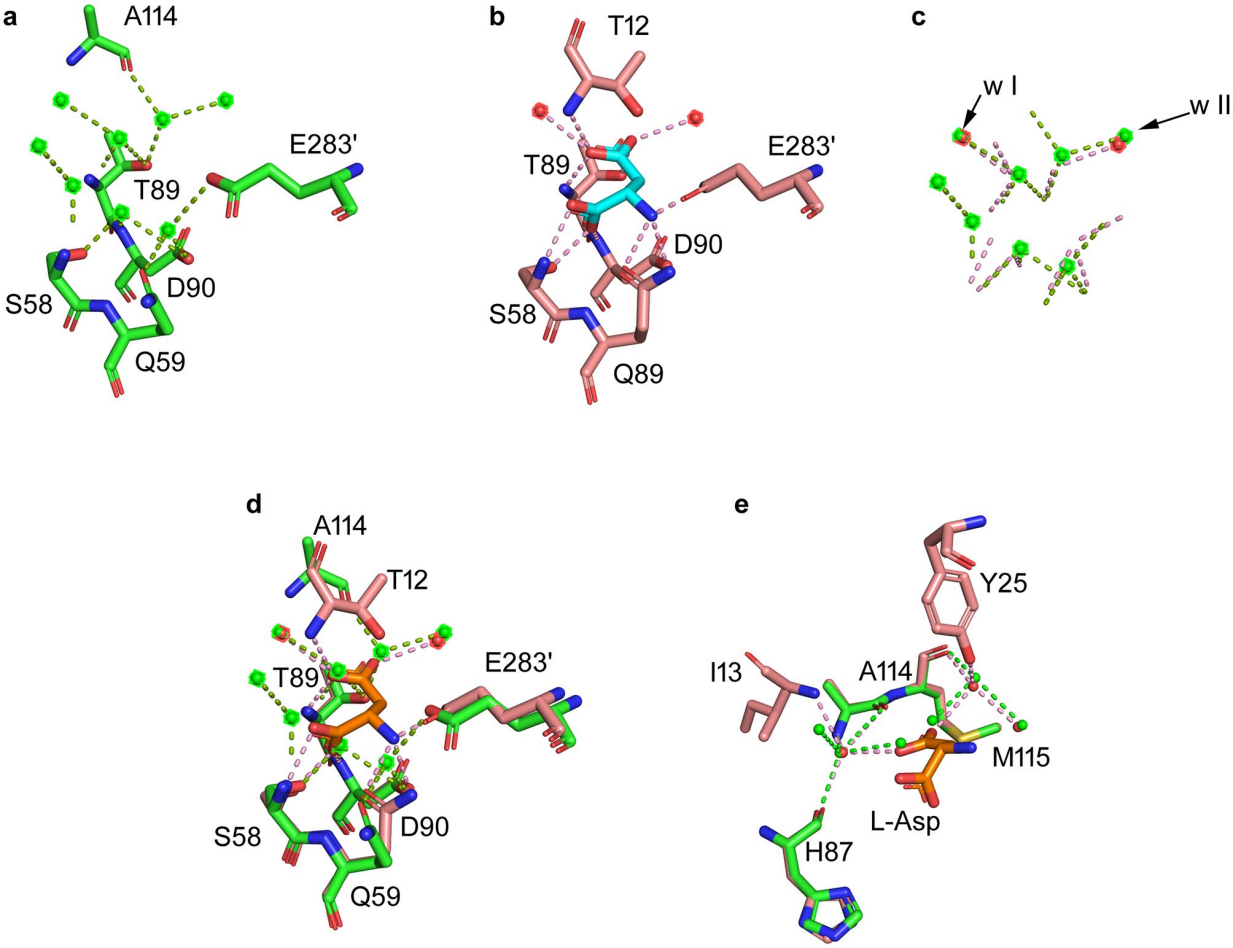
Water molecules displacement in APO-EcAII (green, 6YZI) and EcAII-ASP (pink, 1NNS) active site. (**a**) Coordinated waters in APO-EcAII. (**b**) Coordinated waters in EcAII-ASP. (**c**) Water molecules found in APO-EcAII (green) and EcAII-ASP (red) superposition. Black arrows indicate waters conserved in the two structures. (**d**) Superposition of (**a**) and (**b**). (**e**) Residues involved in contacts with water molecules conserved in APO and EcAII-ASP active site.

Analysis of solvent in the APO-EcAII active site pocket reveals the presence of five highly coordinated water molecules (for reference: 408, 412, 446, 529, 592 in 6YZI chain C) that interact with residues involved in l-Asp binding and are present in all 4 protomers of the ASU. Superposition with the EcAII-ASP structure (PDB ID: 1NNS) shows that the position of the water molecules exactly coincides with that of the l-Asp ligand atoms (Fig. 4d) involved in binding with residues S58, Q59, G88, T89, D90 and E283′ (Fig. 4a,b). As expected, the five superposed waters are not found in the l-Asp-bound structure, where their density is replaced by that of the ligand and the enzyme enters its closed, catalytically competent conformation. Interestingly, upon loop closing on the binding pocket, only one extra bond is evident between the ASN substrate OD1 (PDB: 6V2B) and T12 N atom; all the other bonds are already in place when water molecules occupy the enzyme binding pocket (Fig. 4e).

Two further water molecules interacting with l-Asp OD1 and OD2, respectively, are present in the APO-EcAII [named water I (416) and water II (521)] and EcAII-ASP (PDB ID: 1NNS) structures in exactly the same positions (Fig. 4c). The role of these water molecules can be relevant in the enzyme catalysis, as they are highly conserved in all crystalline structures of wild type EcAII. In Y25F EcAII mutant (PDB ID: 1HO3) water I is absent, very likely because of the strong hydrophobicity of the F25 side chain, and, consequently, the mutant has highly reduced catalytic rate, while, interestingly, its affinity for ASN as a substrate is unchanged^22^. Analysis of EcAII acyl-intermediates (PDBs: 4ECA, 6V2C, 6V2G, 6V27, 6V28), suggests that neither of the two water molecules is directly involved in the first part of the catalytic process, as they are conserved also in the intermediate state and acquire the same sets of contacts described for the l-Asp-bound wild-type. Accordingly, it is possible to speculate that water I is involved in the second step of catalysis, where the enzyme-acyl intermediate is solved by the intervention of a water molecule that acts as a second nucleophile. In asparaginases, the active site tyrosine can either act as a chaperon, to carry a water molecule in position I during loop closure^25^, or as a water activator, to facilitate the nucleophile attack that solves the acyl intermediate^12^. The fact that water I is already present in the APO-EcAII active site, when the loop is still in the open conformation, proves a role for Y25 as proton conveyor onto water I, which is also supported by the reduction in activity, and not in affinity for the substrate, of the Y25F mutant, where activation of water I would be impaired for the lack of the hydroxyl group of the Y25 side chain.

Moreover, K162M acyl intermediate structure (PDB ID: 6V24) presents a water molecule in the same position of l-Asp OD1, similarly to the APO structure, suggesting that waters in the binding pocket are replaced by the substrate upon its proper binding. The same water is not present in the T89V mutant acyl intermediate (PDB ID: 4ECA), as it is, indeed, coordinated by the T89 OH group, that is missing in T89V.

### Global dynamics in response to the ligand

**Figure 5:**
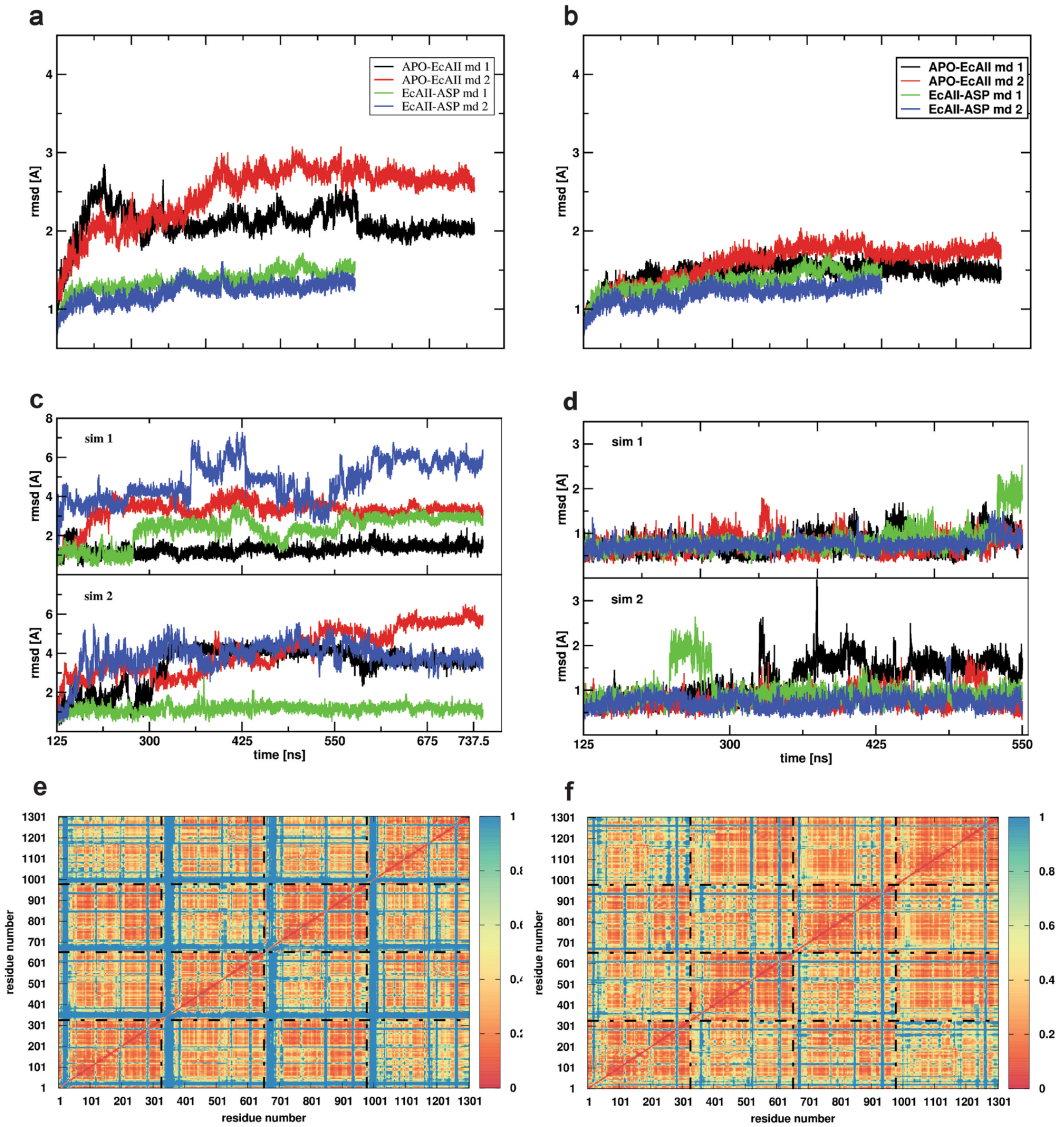
Dynamics of APO and EcAII-ASP. Root-mean-square deviation of atomic positions along the simulation: (**a**) on all Cα of l-asparaginase without (APO) and with l-Asp present in the catalytic site. (**b**) Same as a without the Cα that belong to the catalytic loop. (**c**) Catalytic loop without l-Asp in the catalytic site (APO form). (**d**) Same as (**c**) with l-Asp in the catalytic site (HOLO form). (**e**) and (**f**) Distance Fluctuation matrices for EcAII with (**f**) and without (**e**) l-Asp in the catalytic site.

We next set out to characterize the dynamics of the protein at a global and local level by means of all-atom MD simulations. Calculations were carried out on the APO-EcAII and EcAII-ASP forms of the protein. Figure 5a–d show the RMSD relative to the starting X-ray structure as a function of time. The RMSD plots of the whole protein are consistently higher in the case of the unbound protein compared to the bound one (Fig. 5a). To investigate the origin of the dynamic difference in the APO-EcAII vs. EcAII-ASP states, we first re-calculated the RMSD of the whole protein, excluding residues of the catalytic loop (from residue 8 to residue 32) from the calculation (Fig. 5b). A dramatic drop in the RMSD values for the nonbonded (APO-EcAII) states is immediately evident, indicating that the main source of divergence for RMSD can be expectedly traced to the dynamics of the catalytic loop. In a complementary way, the evaluation of the RMSD values only for the loop region, separately for each monomer in the tetramers was carried out (Fig. 5c,d). Interestingly, in the bound state, the presence of l-Asp appears to minimize the motions of the loops of all four tetramers (Fig. 5d). The analysis of the RMSD of the catalytic loop region (Fig. 5c) corroborates this observation, showing that the presence of the ligand minimizes the flexibility of all catalytic loops. In this picture, the presence of the ligand (l-Asp) induces the loop to move towards the rigid part of the active site with the most affected residues belonging to region 16–32.

Next, the internal dynamics of the APO-EcAII and EcAII-ASP states were compared, for the purpose of identifying possible coordination patterns between proximal and distant protein regions that can be affected/generated by ligand binding. To this end, we calculated the variance of the inter-residue distance of all residue pairs, resulting in an *N* × *N* matrix (where N is the number of residues) called Distance Fluctuation matrix (Fig. 5e,f). Low values of this parameter identify protein subdomains that are mechanically coordinated during the dynamics, and vice-versa (Fig. 5e,f).

In general, the comparison of the matrices shows a similar block character, which reflects the tetrameric organization of the protein. Closer inspection indicates that the bound form is characterized by a substantially higher and more diffuse degree of internal coordination, corresponding to a global rigidification, caused mainly by the formation of the contacts between l-Asp and the catalytic loops. Interestingly, the effect of the ligand appears thus to diffuse long range from the binding site to the whole structure.

Complementary to this analysis, to further characterize the modulation of the global dynamics of the protein in response to the presence of a ligand in the active site, we calculated all the hydrogen bonds (HBs) formed at the inter-protomer surface of both the APO-EcAII and EcAII-ASP to expose possible variations due the presence of the substrate in the active site. The presence of the l-Asp ligand in the active site modulates the inter chain interactions mediated by the HBs, significantly modifying both the residues and the structures involved. However, only one residue, S23, part of the active site catalytic loop, is involved in H-bonds stabilized by l-Asp binding, while the other residues belong to coiled regions close to, but out of, the enzyme binding pocket. Four out of 10 stabilized residues belong to the protomer C-domain, in particular to the region in proximity of the catalytic E283′ residue, pointing at an inter monomers stabilization induced by the ligand.

Analysis of the contacts formed during the MD simulation between the active site flexible loop residues and the rest of the protein was carried out next. A residue of the catalytic loop is considered to establish a “stable” contact with other residues of the protein if the distance between their Cα atom is lower than 6 Å for more than 30% of the total of sampled conformations (total conformations obtained by combining the data from the two simulation runs for each system). In Table 1, we report the most important contacts established in APO-EcAII and EcAII-ASP during the MD simulation according to this criterion. Overall, only 16 (bold) out of the 53 EcAII-ASP contacts are maintained in the APO-EcAII form. Interestingly, contacts between the active site flexible loop and the coil region comprised between residues 115–120 are present only in the APO-EcAII structure and are lost in EcAII-ASP. These interactions can have a role in loop stabilization in the enzyme open conformation.

**Table 1:** H-bonds (HBs) comparison in APO-EcAII and EcAII-ASP. In bold HB conserved in APO-EcAII and EcAII-ASP, in italics HBs involving residues in the same region both in APO-EcAII and EcAII-ASP, and, in bolditalics HBs different in APO-EcAII and EcAII-ASP.

HOLO				APO				
1st Res name	1st Res number	1st Res name	1st Res number	2nd Res name	2nd Res number	2nd Res name	2nd Res number	
**GLY**	**16**	**PRO**	**117**	**GLY**	**16**	**PRO**	**117**	*G16*
				*GLY*	*16*	*SER*	*118*	
**GLY**	**16**	**THR**	**119**	**GLY**	**16**	**THR**	**119**	
				***GLY***	***17***	***THR***	***119***	***G17***
				***GLY***	***17***	***ALA***	***38***	
				***ASP***	***18***	***THR***	***119***	***D18***
				***SER***	***19***	***THR***	***119***	***S19***
				***SER***	***19***	***SER***	***120***	
				*ALA*	*20*	*THR*	*119*	**A20**
**ALA**	**20**	**SER**	**120**	**ALA**	**20**	**SER**	**120**	
**ALA**	**20**	**MET**	**121**	**ALA**	**20**	**MET**	**121**	
**THR**	**21**	**SER**	**120**	**THR**	**21**	**SER**	**120**	*T21*
				***THR (A)***	***21***	***GLY (B)***	***17 (B)***	
**LYS**	**22**	**ASN**	**184 (+ chain)**	**LYS**	**22**	**ASN**	**184**	**K22**
				***SER***	***23***	***ARG***	***116***	***S23***
				***SER***	***23***	***SER***	***120***	
				***ASN***	***24***	***ARG***	***116***	***N24***
				***ASN***	***24***	***GLY***	***15***	
***ASN***	***24***	***ALA***	***282 (*+ *chain)***					
***ASN***	***24***	***GLU***	***283 (*+ *chain)***					
				***TYR***	***25***	***MET***	***115***	***Y25***
				***TYR***	***25***	***ARG***	***116***	
				***TYR***	***25***	***PRO***	***117***	
*TYR*	*25*	*GLY*	*16*	*TYR*	*25*	*GLY*	*15*	
*TYR*	*25*	*GLY*	*17*					
				***TYR***	***25***	***ALA***	***38***	
				***THR***	***26***	***PRO***	***117***	*T26*
				***THR***	***26***	***SER***	***118***	
**THR**	**26**	**GLY**	**15**	**THR**	**26**	**GLY**	**15**	
**VAL**	**27**	**GLY**	**11**	**VAL**	**27**	**GLY**	**11**	*V27*
				***VAL***	***27***	***SER***	***118***	
**VAL**	**27**	**THR**	**12**	**VAL**	**27**	**THR**	**12**	
**VAL**	**27**	**GLY**	**15**	**VAL**	**27**	**GLY**	**15**	
				*VAL*	*27*	*ALA*	*14*	
***VAL***	***27***	***GLY***	***57***					
				***VAL***	***27***	***ASN***	***34***	
*GLY*	*28*	*GLY*	*10*	*GLY*	*28*	*THR*	*12*	***G28***
**GLY**	**28**	**GLY**	**11**	**GLY**	**28**	**GLY**	**11**	
**GLY**	**28**	**ALA**	**14**	**GLY**	**28**	**ALA**	**14**	
**GLY**	**28**	**GLY**	**15**	**GLY**	**28**	**GLY**	**15**	
				***GLY***	***28***	***SER***	***118***	
***GLY***	***28***	***ASN***	***55***					
***GLY***	***28***	***ILE***	***56***					
*LYS*	*29*	*ALA*	*14*	*LYS*	*29*	*GLY*	*11*	**K29**
*LYS*	*29*	*GLY*	*15*					
*VAL*	*30*	*ALA*	*14*	*VAL*	*30*	*GLY*	*11*	**V30**
				*VAL*	*30*	*THR*	*12*	
				*VAL*	*30*	*GLY*	*15*	
				***GLY***	***31***	***GLY***	***10***	***G31***
				***GLY***	***31***	***GLY***	***11***	
				***GLY***	***31***	***THR***	***12***	
				*VAL*	*32*	*GLY*	*11*	**V32**
				*VAL*	*32*	*THR*	*12*	
**VAL**	**32**	**GLY**	**50**	**VAL**	**32**	**GLY**	**50**	
				*VAL*	*32*	*GLN*	*52*	
**VAL**	**32**	**ALA**	**8**	**VAL**	**32**	**ALA**	**8**	
***THR***	***12***	***ALA***	***114***	***THR***	***12***	***LEU***	***35***	***T12***
***THR***	***12***	***PRO***	***117***					
**THR**	**89**	**GLY**	**113**	**THR**	**89**	**GLY**	**113**	**T89**

## Conclusions

In this paper, we describe for the first time the complete crystallographic structure of *E. coli* type II l-asparaginase in its ligand-free open conformation. Moreover, we also performed for the first time molecular dynamics studies on the open conformation of the enzyme.

Comparison of APO-EcAII and EcAII-ASP structures confirmed that residues belonging to the N-domain active site loop (e.g., residues 9–31) are the most affected by structural changes happening upon substrate binding. Molecular Dynamics simulations are consistent with this experimental observation, being residues 8–32 the region with the highest RMSD divergence. Interestingly, analysis of the APO and l-Asp-bound internal dynamics showed that, even though major structural rearrangements are localized at the active site flexible loop, a global structural stabilization is observed in the l-Asp-bound protein, indicating a long range effect of the loop stabilization in the enzyme closed conformation. Such a stabilization affects also inter-protomers interactions in the protein intimate dimer and largely involves residues belonging to the C-domain component of the active site (e.g., E283 region). Interestingly, and relevant for the molecule engineering, most of the residues involved in catalysis and substrate binding that are external to the active site flexible loop are coordinated and properly positioned in the active site rigid part already in the enzyme open conformation, along with water molecules involved in the reaction.

In conclusion, the collected data and the availability of a complete crystallographic structure of APO-EcAII allow a better understanding of the enzyme catalytic mechanism and dynamism, contributing key information for the molecule engineering aiming at improving its efficacy as an anti-cancer drug.

## Materials and methods

### Protein expression and purification

Recombinant *Escherichia coli* type II l-asparaginase (EcAII) was produced in recombinant form as described in Maggi et al.^26^. Briefly, protein overexpression was obtained by the autoinducing method using *E. coli* BL21(DE3) *Δans*A*/Δans*B as a host strain. Protein purification was obtained by immobilized metal affinity (HisTrap, GE Healthcare) and anionic exchange chromatography (HiTrap Q, GE healthcare). Protein to be crystallized was buffer exchanged on column (HiTrap Desalting, GE Healthcare) against 50 mM HEPES (4-(2-hydroxyethyl)-1-piperazineethanesulfonic acid), pH 7.4. Protein solution pure to homogeneity was concentrated to 2.5 mg/ml and used for crystallization. Multiple prismatic crystals were obtained in condition n° 19 of Molecular Dimensions structure screening 2 (MD-02). The condition was further optimized and modified as follows: 0.1 M Sodium HEPES pH 8.5, 10% w/v PEG 8000, 5% v/v Ethylene glycol. Single prismatic crystals were obtained in a sitting drop setting at 21 °C after 4–5 days and mounted on nylon loops in a cryoprotectant solution containing the crystallization condition added with 25% v/v glycerol.

### Data collection and refinement

X-ray diffraction data were collected at the European Synchrotron Research Facility (ESRF, Grenoble, France) at beamline ID23-2. The data were processed using iMOSFLM^27^ and SCALA^28^. The asymmetric unit (ASU) content was estimated by Matthews coefficient. The structure was solved by molecular replacement with Phaser^29^ and using a single monomer missing residues 10–31 derived from the deposited structure of EcAII (PDB ID: 3ECA) as a probe. The structure was refined alternating cycles of refinement with Phenix^30^ (real space, reciprocal space, individual B factors and occupancy) and manual rebuilding based on electron density maps by Coot^31^. Translation/Libration/Screw (TLS) motion refinement was introduced in the last cycles and water molecules were added automatically by Phenix. 5% of the measured reflections in every data set were reserved for Rfree monitoring during refinement and used for cross-validation. The ASU was rebuilt to contain the two intimate dimers and submitted to the ACHESYM server^32^. The final structure was deposited into the PDB repository with ID: 6YZI. The omit map was calculated in CCP4 SFcheck^33^, using structure factor data and 2 omit cycles. All images were produced by Pymol^19^.

### Molecular dynamics

The crystal structures of the APO-EcAII and EcAII-ASP (PDB ID: 1NNS) protein obtained via X-ray crystallography were used as starting points for all-atom, explicit solvent Molecular Dynamics (MD) simulations. Before starting the MD simulations, the missing loop parts in chain A, B and D for the structure APO-EcAII were modeled using the Modeller software (http://salilab.org/modeller)^34^. Specifically, the following sequence was modeled 16-*GGDSATKSNYTVGKVG-32.*

All crystallographic waters were conserved, and the protonation of acidic or basic groups was selected to be compatible with pH 7.0.

Each of the structures obtained from the procedure described above was solvated in an octahedral box large enough to contain the protein and 1.1 nm of solvent on all sides. The box was filled with explicit water molecules using the TIP3P water model^35^. Counterions were added to yield overall neutral systems. All simulations were run with the Amber 16 suite using the Cuda implementation for GPU.

The minimized systems were then equilibrated at 300 K for 5 ns using Langevin coupling with gamma equal to 1 ps^−1^^36^. After this step, the relaxed systems were simulated in the NPT ensemble at 1 atm using Berendsen coupling algorithms^37^. The full particle-mesh Ewald method was used for electrostatics^38^. The SHAKE algorithm was used to constrain all covalent bonds involving hydrogen atoms^39^. A 2 fs time step and a 10 Å cutoff were used for the truncation of van der Waals nonbonded interactions. The ff14SB force field was used for the protein and the TIP3P model was used for water. Two independent replicas were run for each system of at least 500 ns in length each.

### Distance fluctuation

Protein internal motions were evaluated using a previously introduced distance fluctuation analysis^40^. For each MD trajectory in different bound states, we computed the matrix of distance fluctuations, in which each element of the matrix corresponds to the DF parameter:

$${\text{DF}}_{{{\text{ij}}}} = \left\langle {\left( {{\text{d}}_{{{\text{ij}}}} - \left\langle {{\text{d}}_{{{\text{ij}}}} } \right\rangle } \right)^{{2}} } \right\rangle ,$$

where d_ij_ is the (time-dependent) distance of the C_α_ atoms of amino acids *i* and *j* and the brackets indicate the time-average over the trajectory. This parameter is invariant under translations and rotations of the molecules. The DF matrix can be used to assess the intrinsic flexibility/rigidity of the proteins, and how these properties change upon mutation.

The DF was calculated for any pair of residues during the various trajectories. This parameter characterizes residues that move in a highly coordinated fashion, and it is actually able to reflect the presence of specific coordination patterns. Proteins that are highly coordinated may show enhanced stability.

### Electrostatic potential surface

The procedure used in Pymol (Fig. 3a,b) for the electrostatic potential surface (ESP) calculation was based on the APBS method—Adaptive Poisson–Boltzmann Solver^41^ proposed and described by Baker et al.^42^. The calculated electrostatic potential was then mapped on the surface accessible solvent (SAS), the ESP units were k_b_T/e, where:

- ***k***_***b***_ was the Boltzmann’s constant: k_b_ = 1.38064852 × 10^−23^ J K^−1^
- ***T*** was the temperature in Kelvin
- ***e*** was the electron unit charge in Coulomb: 1.602176634 × 10^–19^ C.

The SAS around the protein were calculated by using a probe sphere with radius 1.4 Å the solvent dielectric constant of the solvent was set to 80, while the solute dielectric constant was set to 1 and the temperature choose for the calculation was 298 K.

## Supplementary Information

Supplementary Information.
