# Supplementary material for: Revealing Escherichia coli type II l-asparaginase active site flexible loop in its open, ligand-free conformation
Source: Sci Rep. 2021 Sep 23;11:18885. doi: 10.1038/s41598-021-98455-1 (PMC8460627; doi:10.1038/s41598-021-98455-1)
Supplement: Supplementary file 1 — Supplementary Information. [file 41598_2021_98455_MOESM1_ESM.docx]

**Supplementary material**


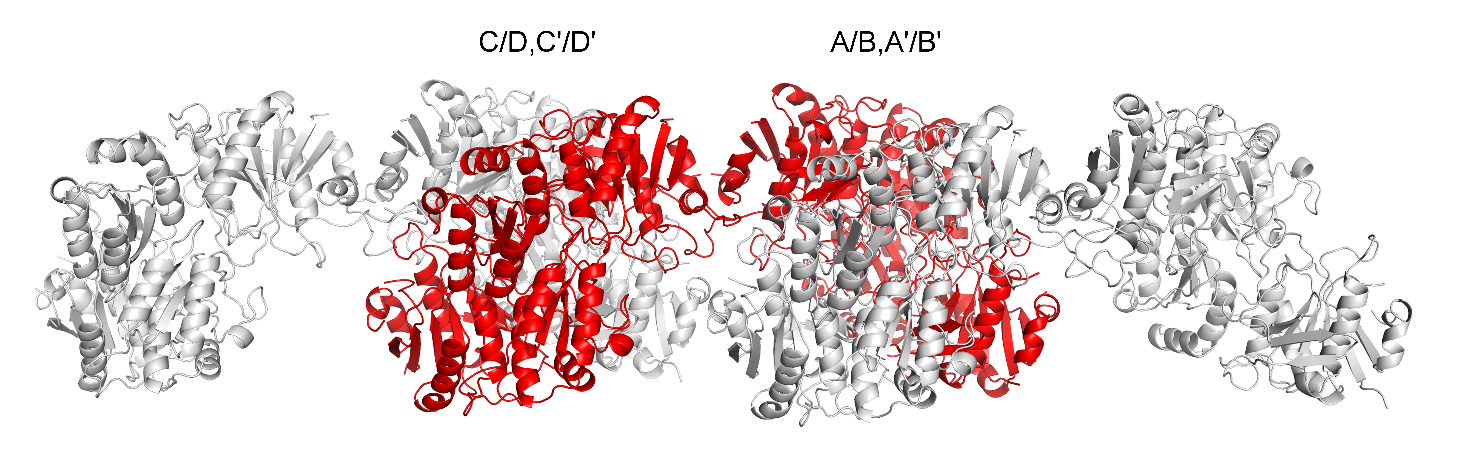


**Supplementary figure 1 Biological unit rebuilt by adjacent ASUs.**


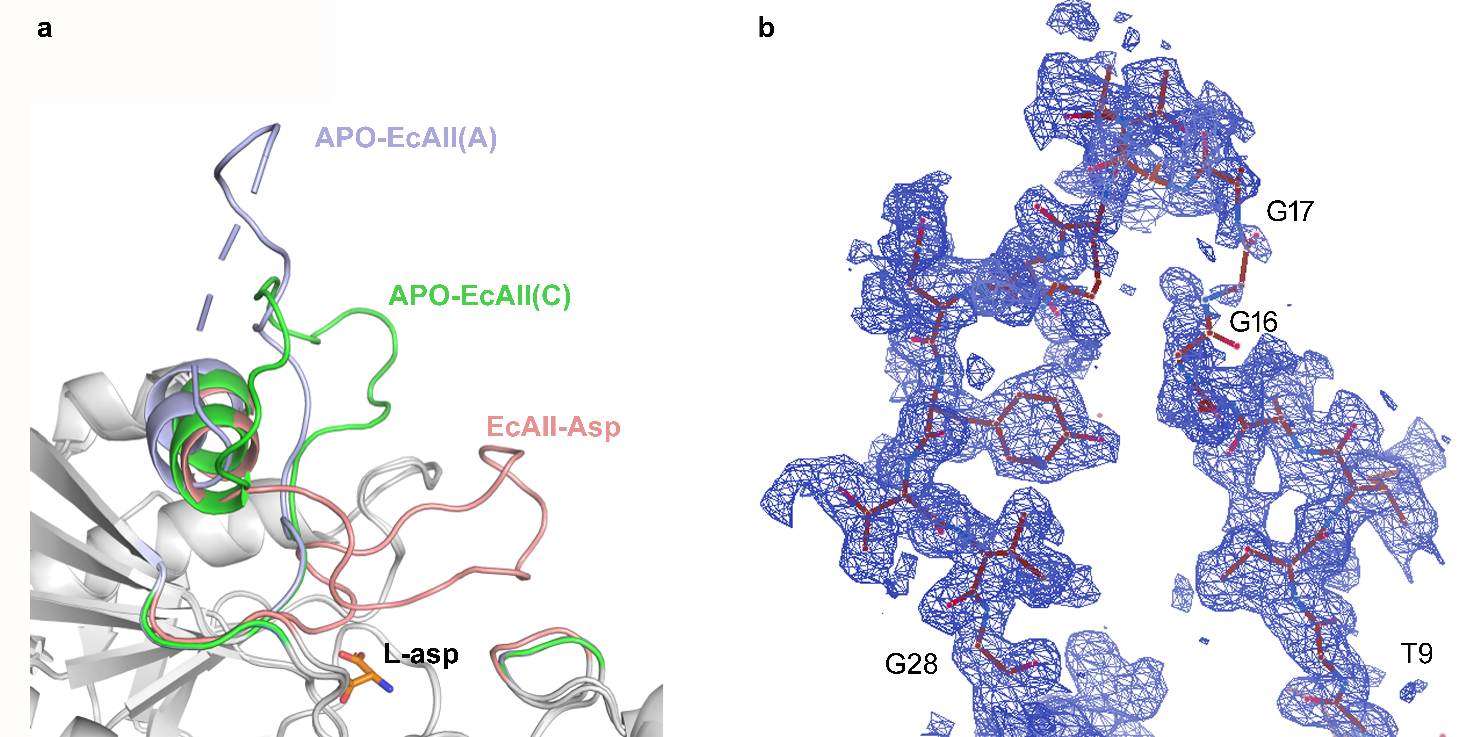


**Supplementary figure 2** Panel a: superposition of the catalytic region of APO-EcAII protomer A (purple) and C (green) and ASP-EcAII (pink). L-Asp ligand is represented as sticks. Only regions different in the three structures are colored. The image is a larger copy of figure 1, panel a. Panel b: OMIT map for the C chain catalytic loop (res. 9-28).


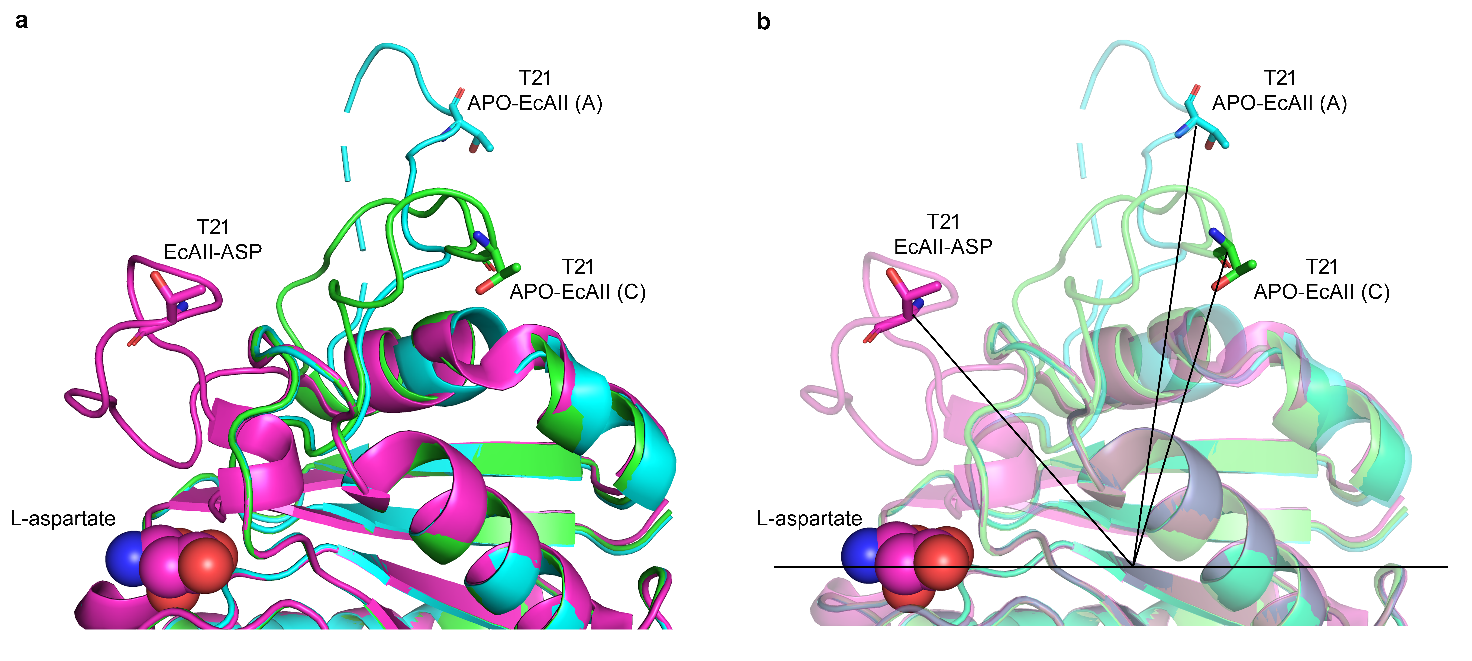


**Supplementary figure 3** Loop orientation in APO-EcAII chain A (purple) and chain C (pink), and in EcAII-ASP (green). T21 is represented as stick in the three monomers. In panel b: schematic representation of the different localizations of residue T21in the three monomers.


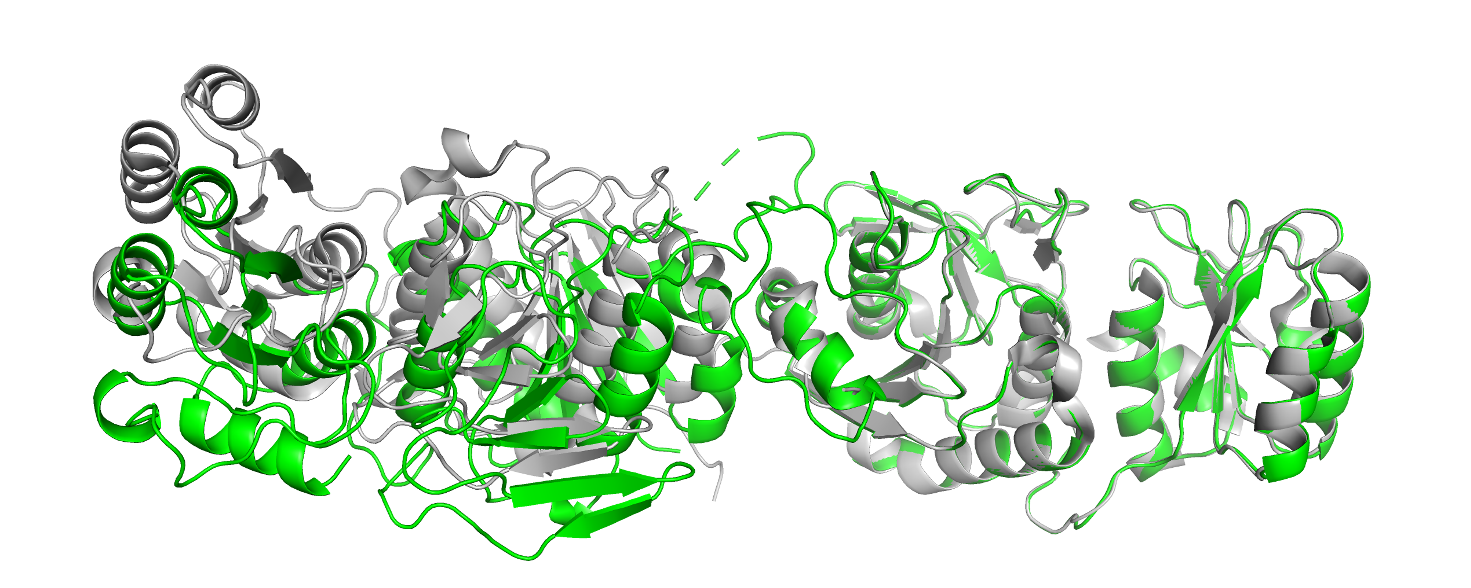


**Supplementary figure 4** The figure represents the superposition of 6YZI adjacent monomers (green) onto corresponding 6V23 ones (gray), adopting chain C as a reference (right monomer in the figure). It is possible to see the displacement of the second monomer (left in the figure) which generates a different crystal environment for the catalytic loops, unable to lock them in place.

**Supplementary table 1.** Crystallographic data and statistics for APO-EcAII. High resolution shell is shown in parenthesis.

|  | 6YZI |
| --- | --- |
| Data collection |  |
| Space group | C 2 |
| Cell dimensions |  |
| *a, b, c* (Å) | 152.240 62.618 143.620 |
| α, β, γ (°) | 90.00 118.10 90.00 |
| Resolution (Å) | 48.4-1.59(1.65-1.59) |
| *R_merge_* | 0.063(1.321) |
| Mean I/σI | 8.3(1.4) |
| Completeness (%) | 100(100) |
| Redundancy | 6.8(6.8) |
|  |  |
| Refinement |  |
| Resolution (Å) | 1.6 |
| No. of reflections | 155436 |
| *R_work_ / R_free_* | 0.16/0.19 |
| No. atoms |  |
| Protein | 18824 |
| Ligand | 0 |
| Water | 1308 |
| *B*-factors (Å^2^) |  |
| Protein | 17.63 |
| Ligand | n.a. |
| Water | 29.14 |
| R.m.s. deviations |  |
| Bond length (Å) | 0.0076 |
| Bond angles (°) | 0.86 |
